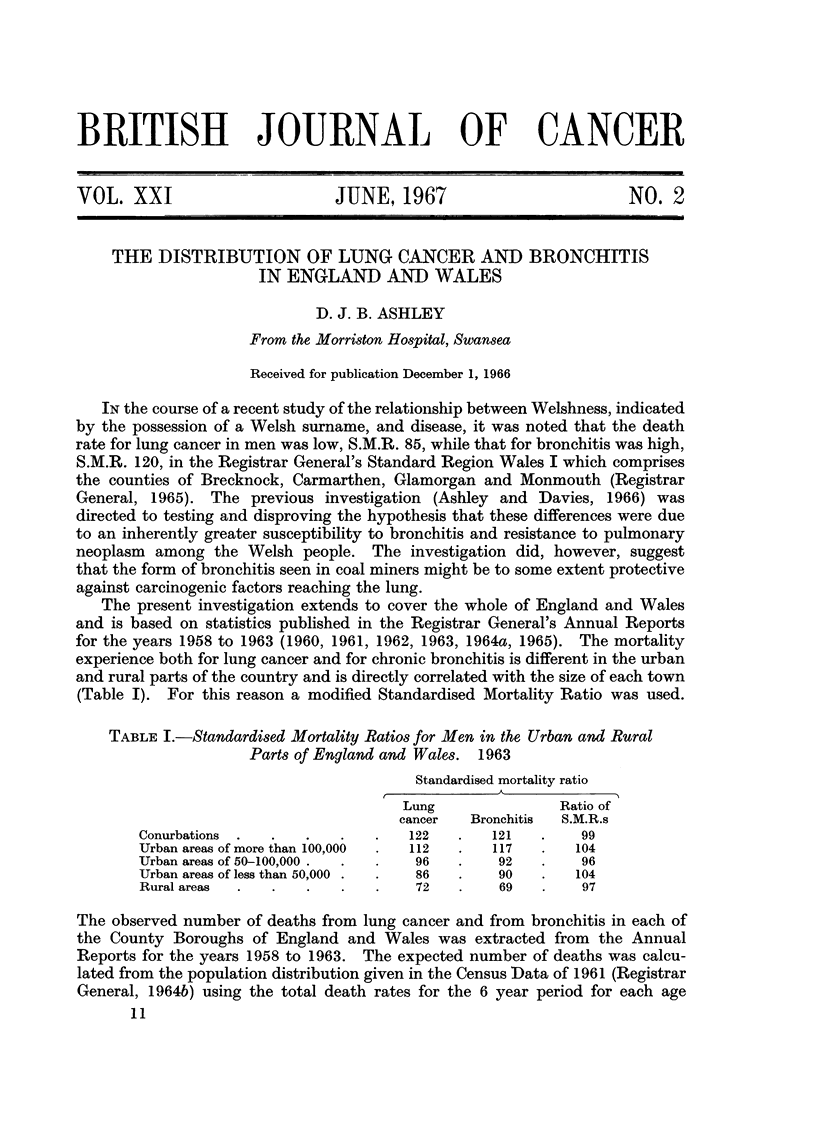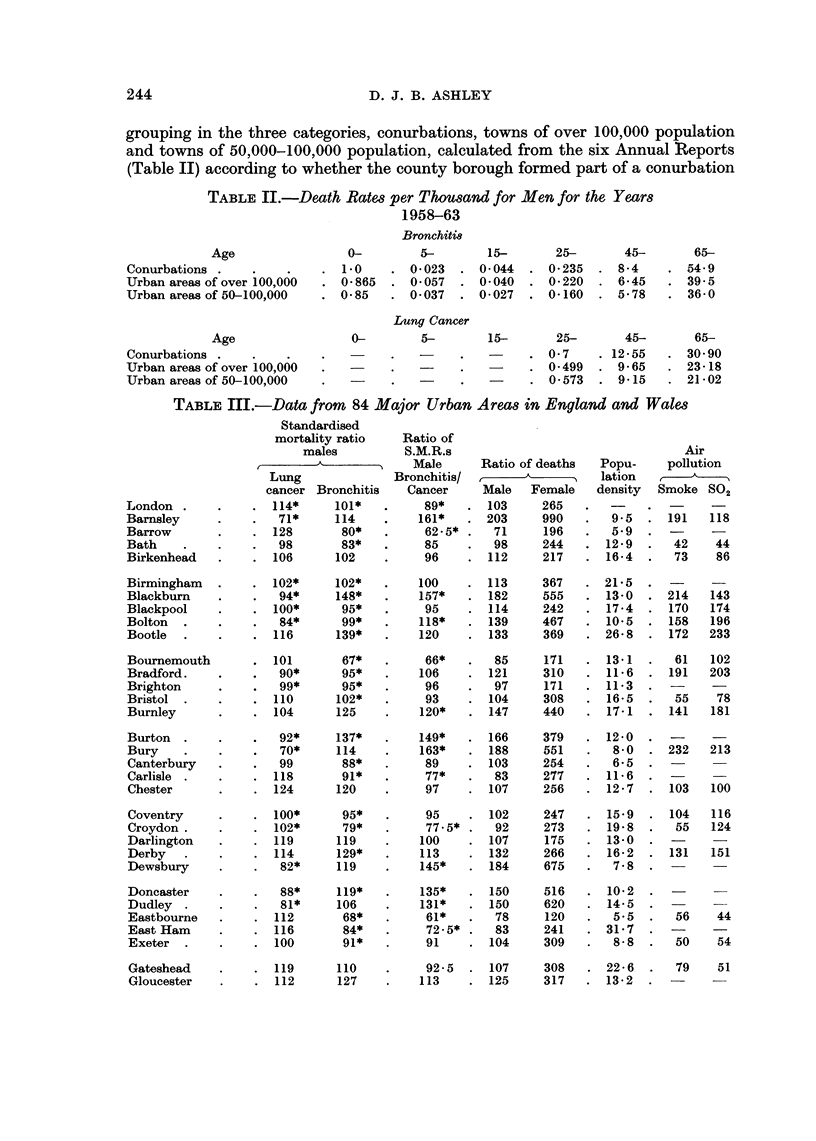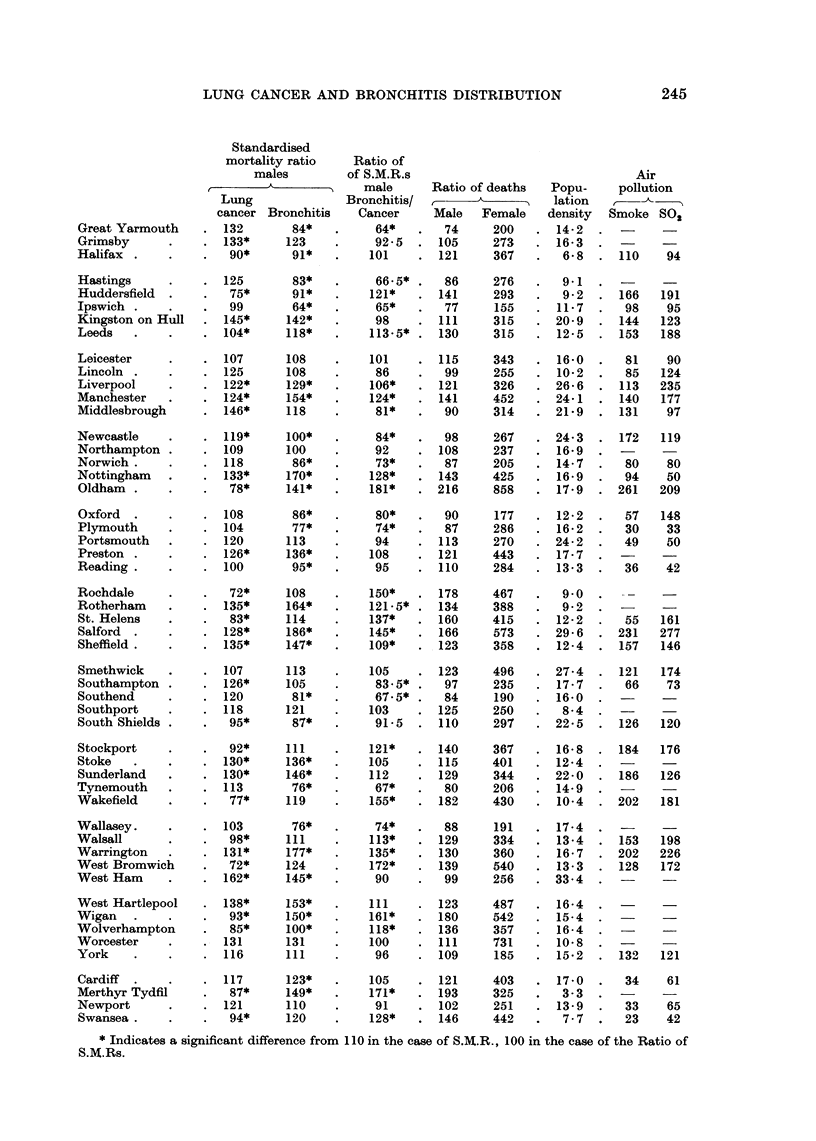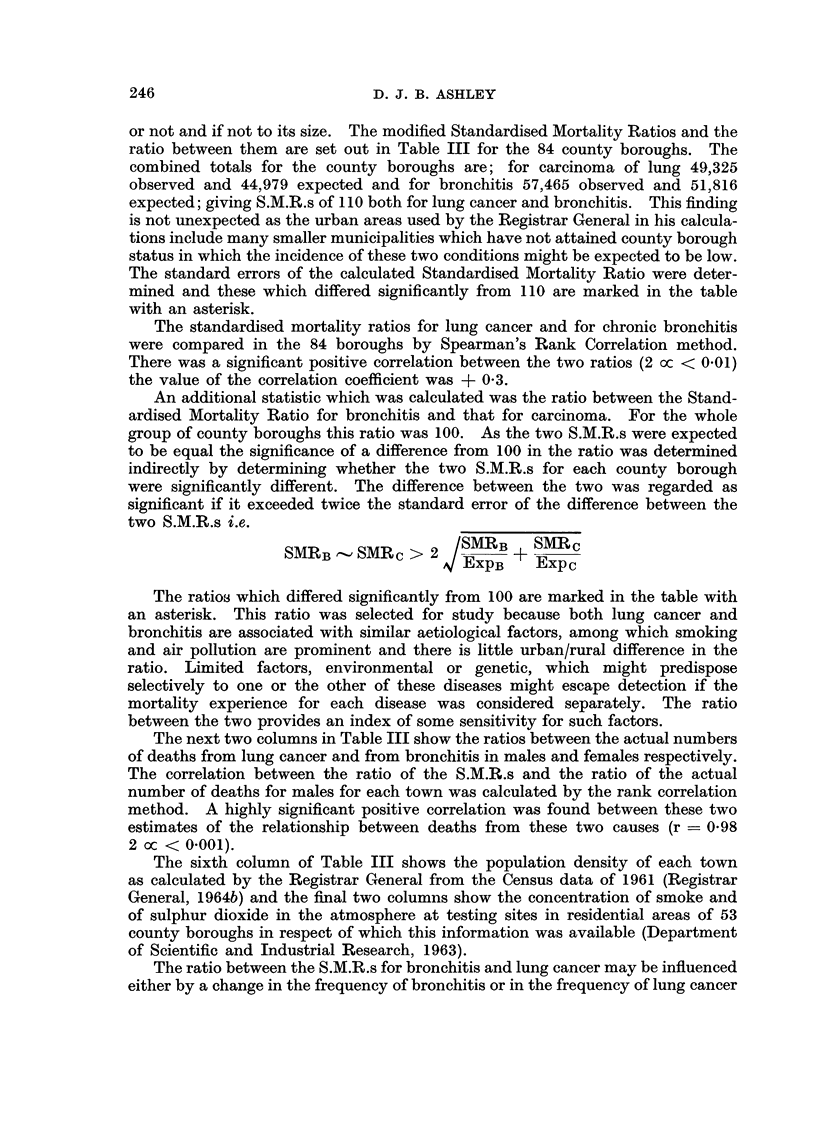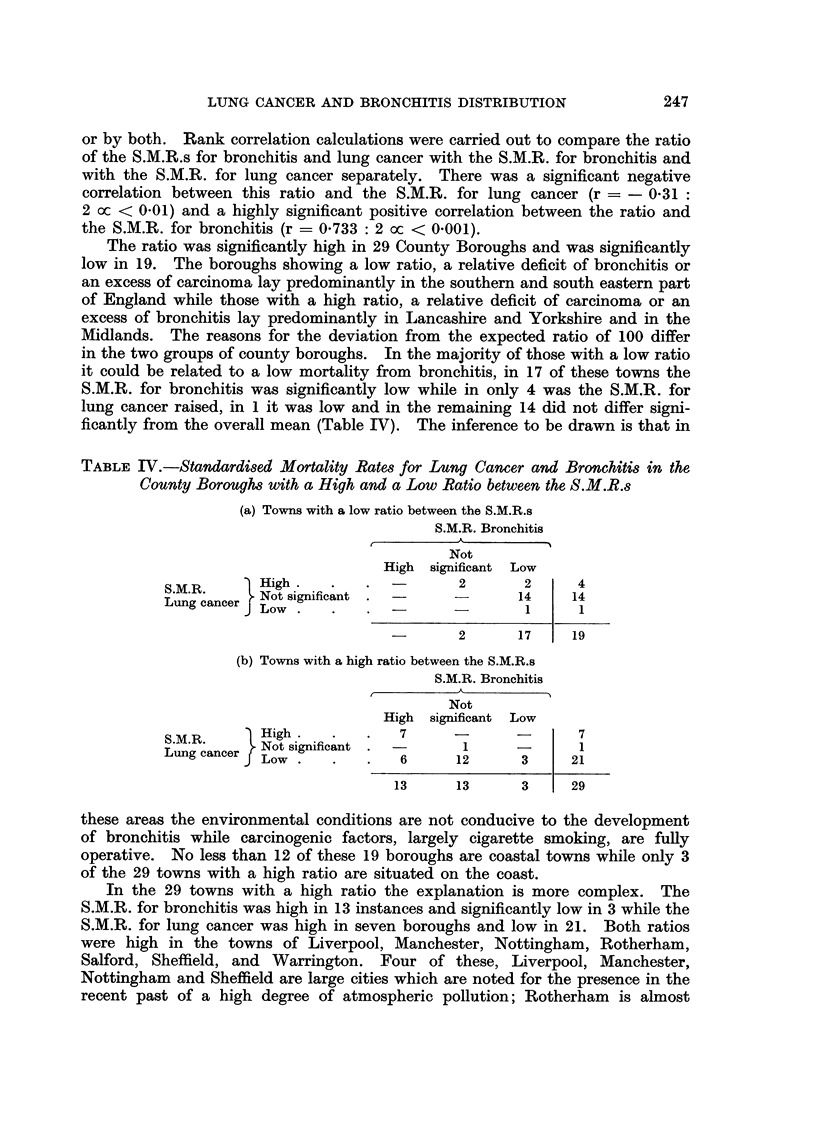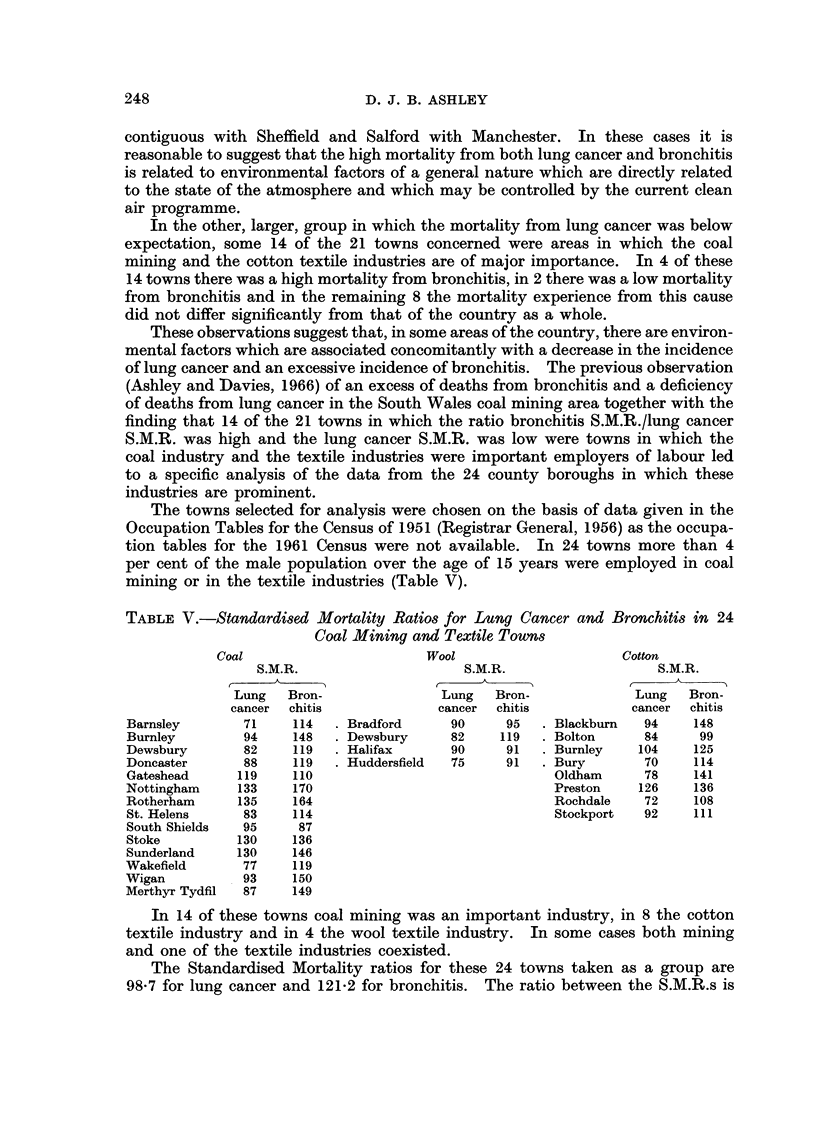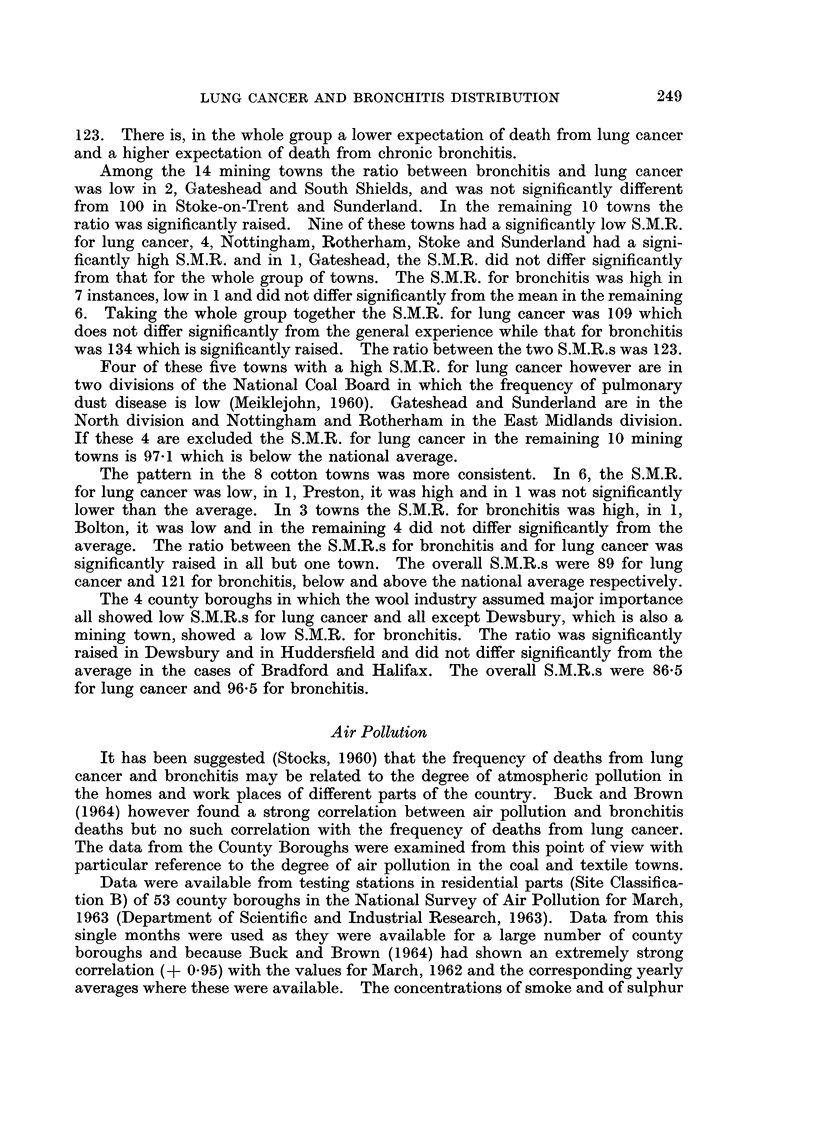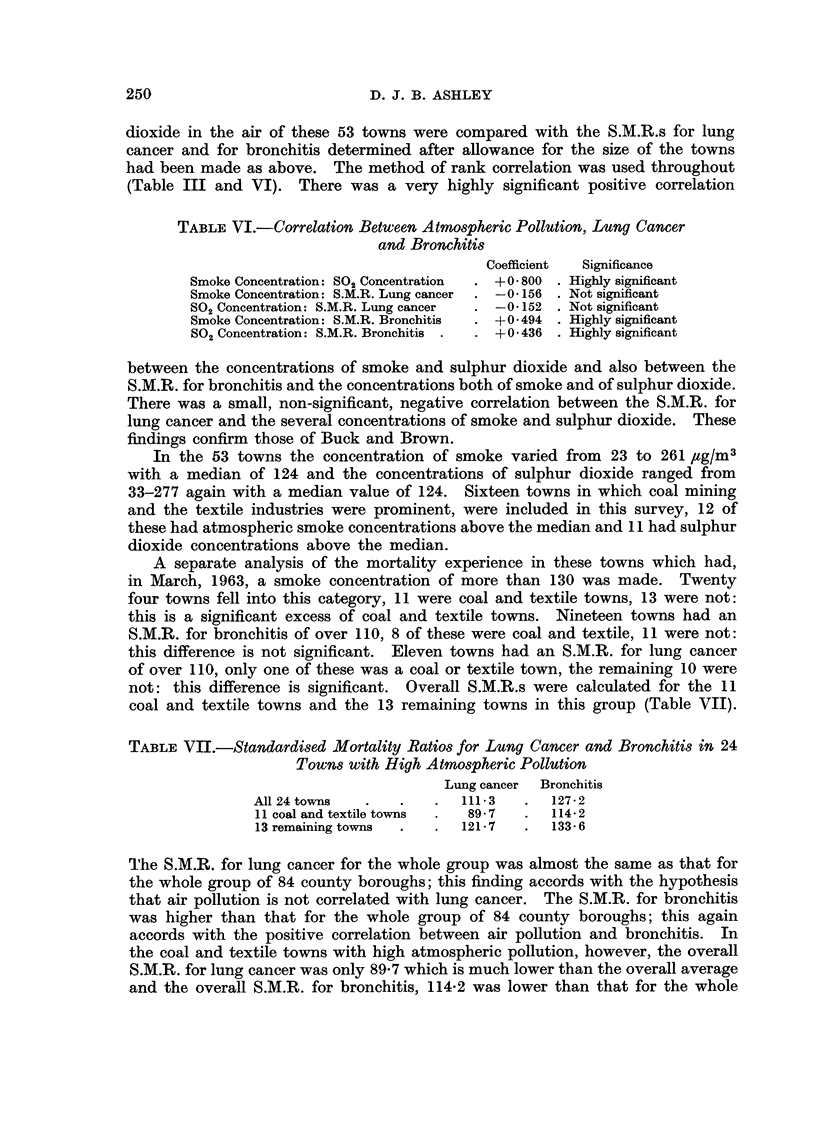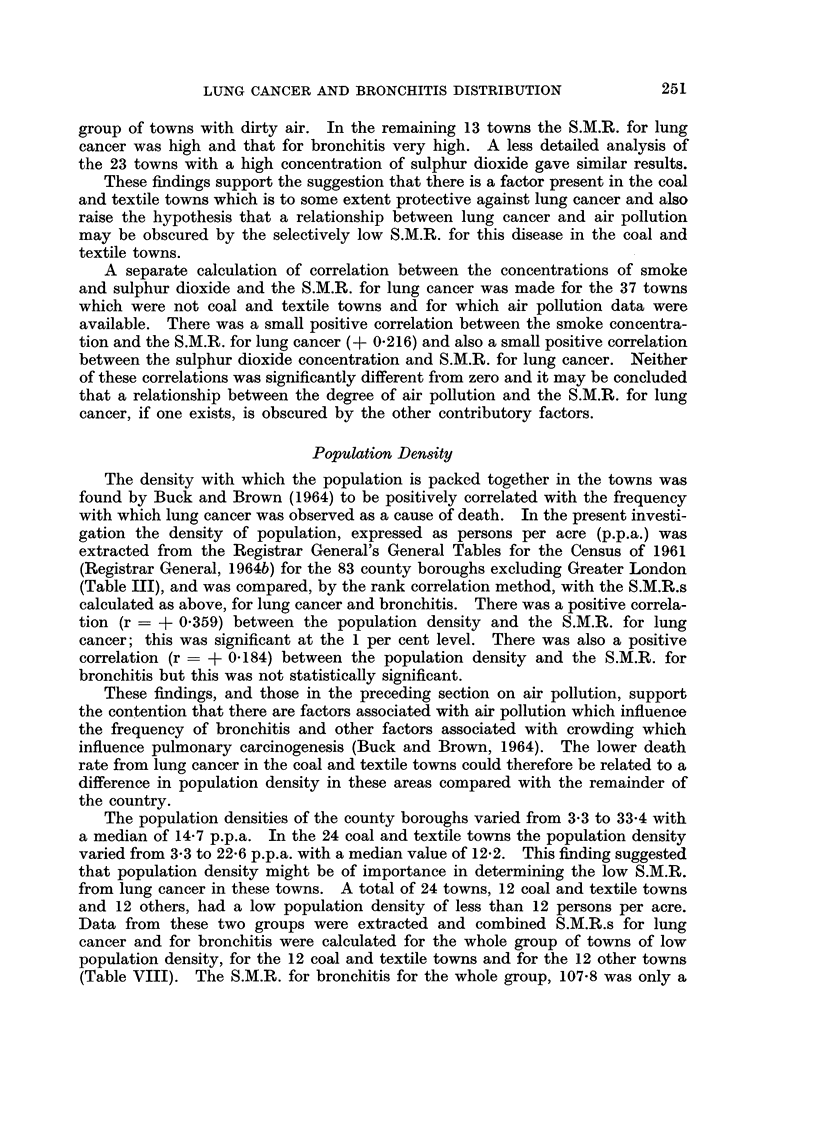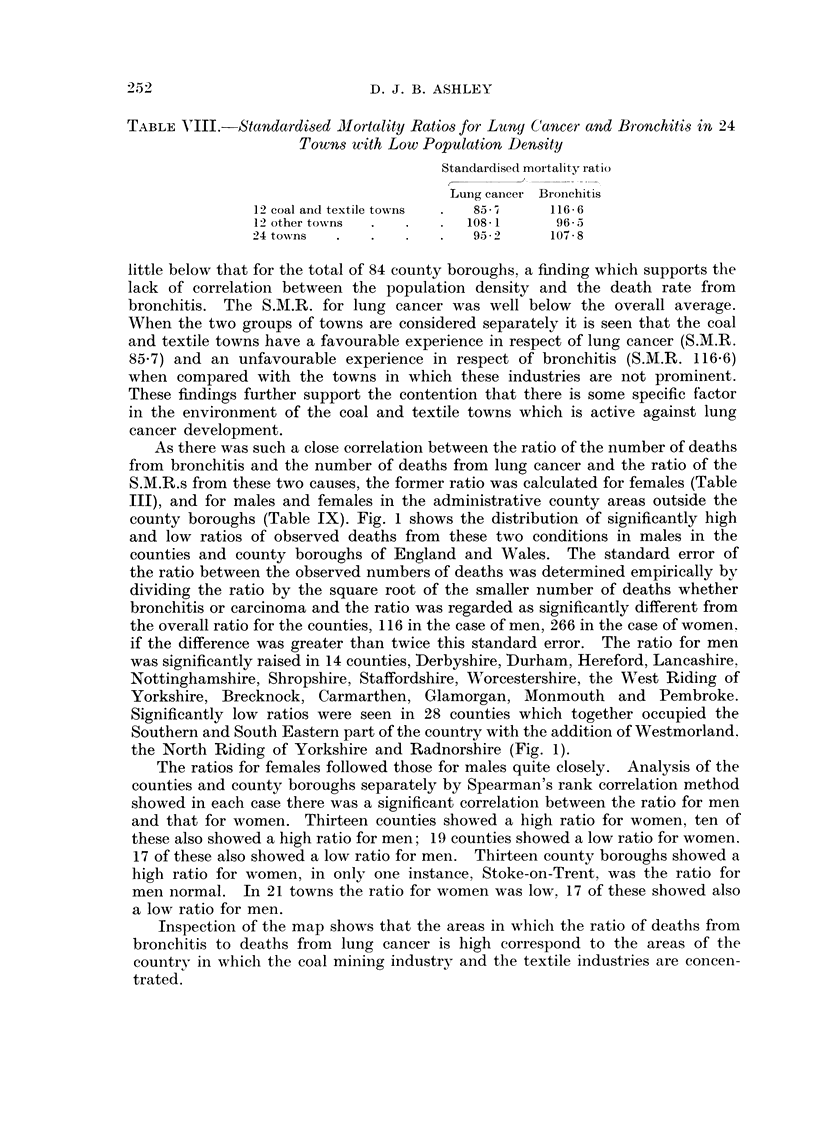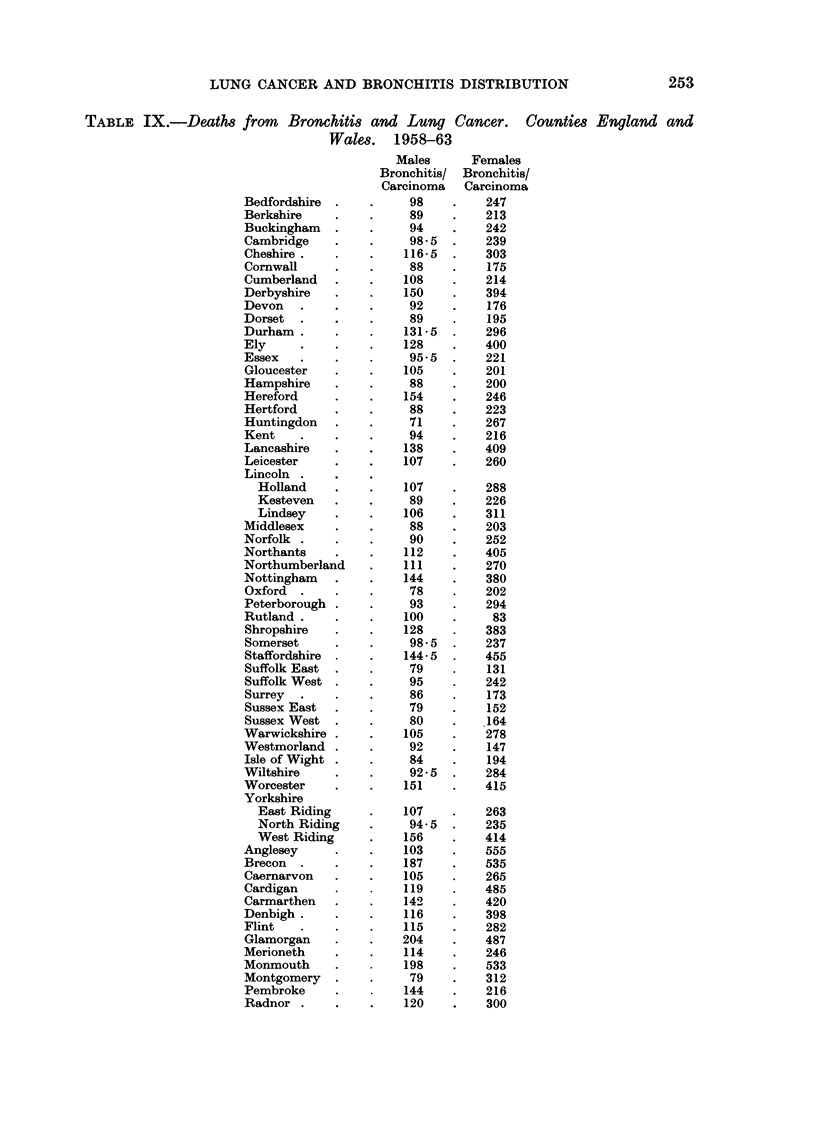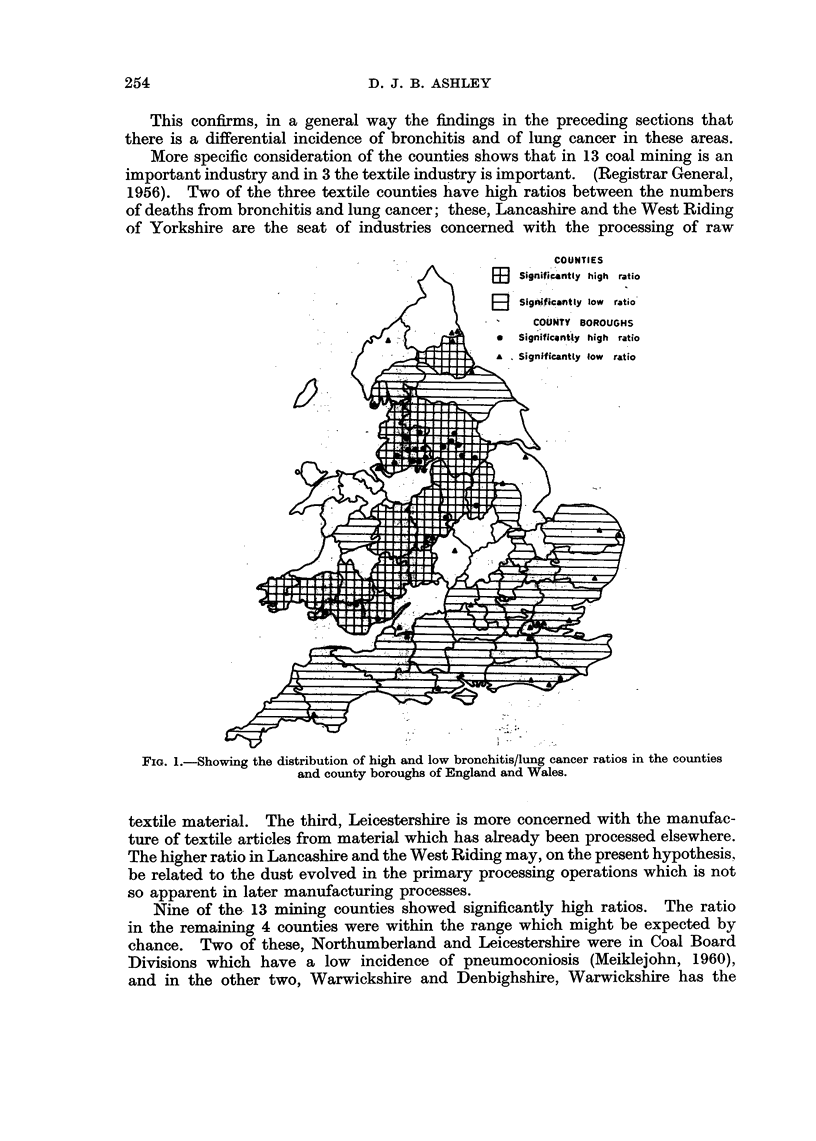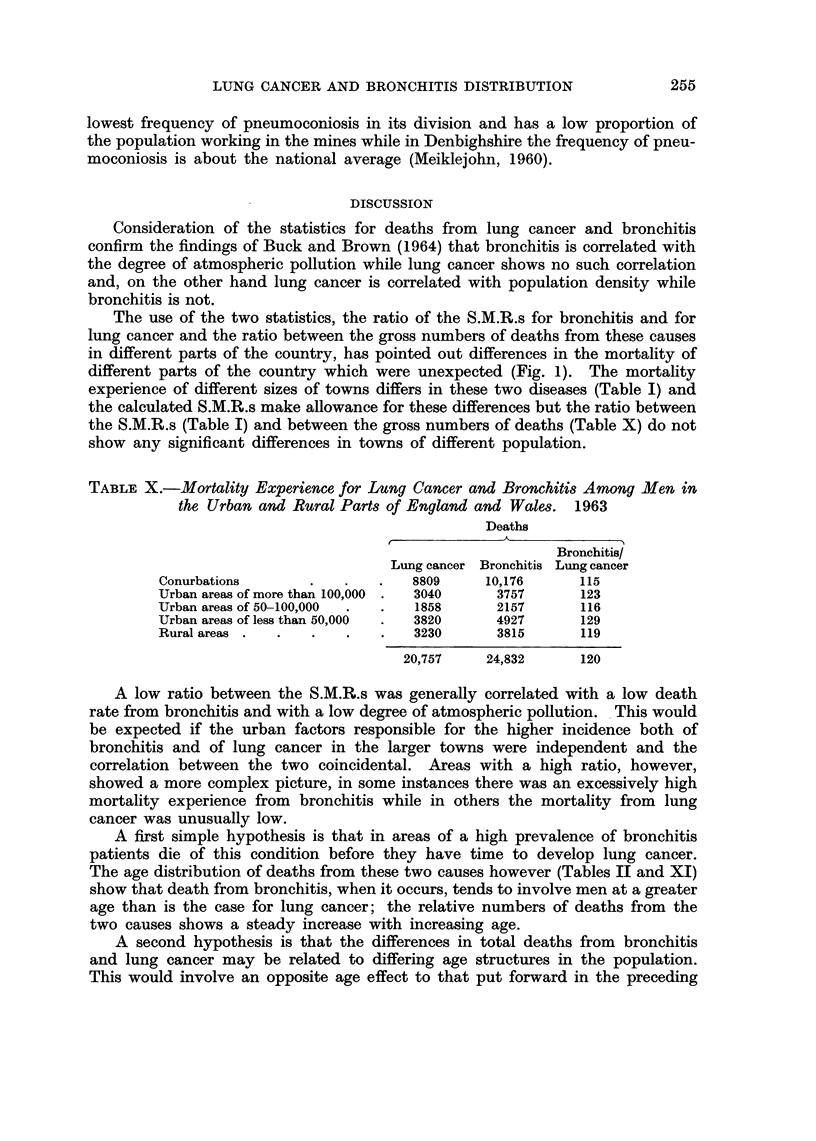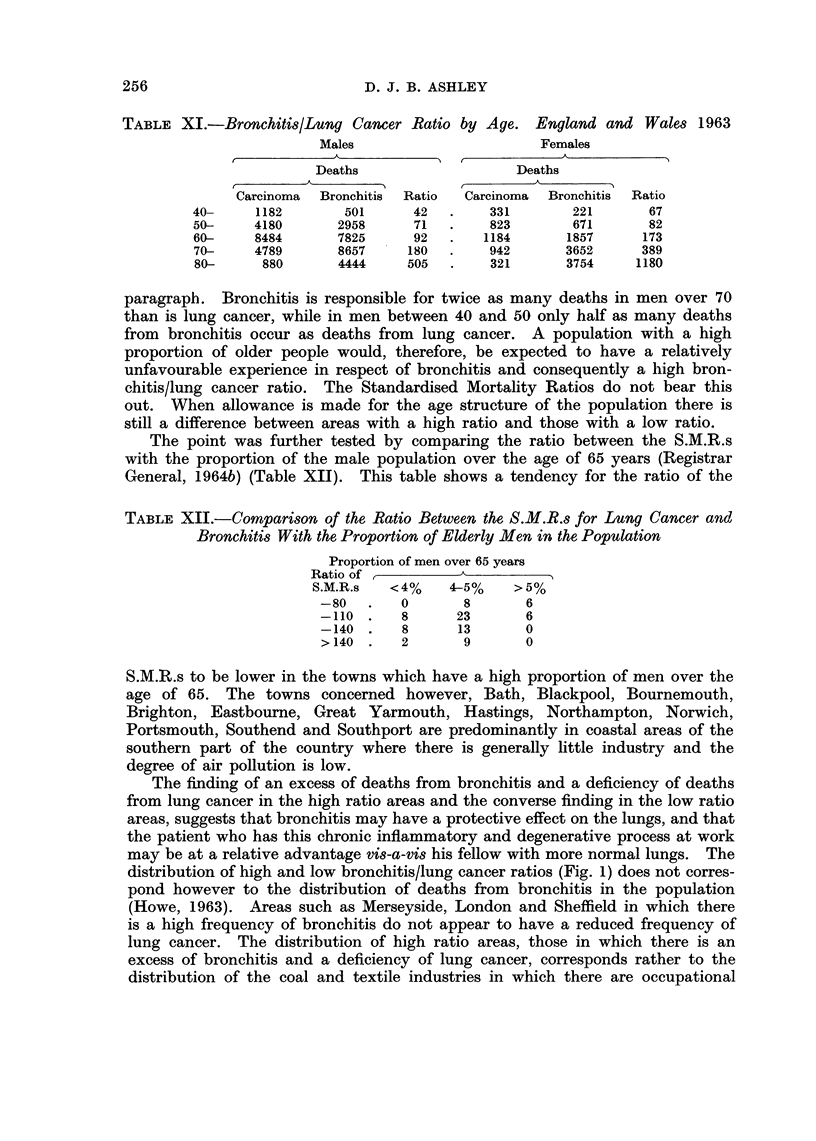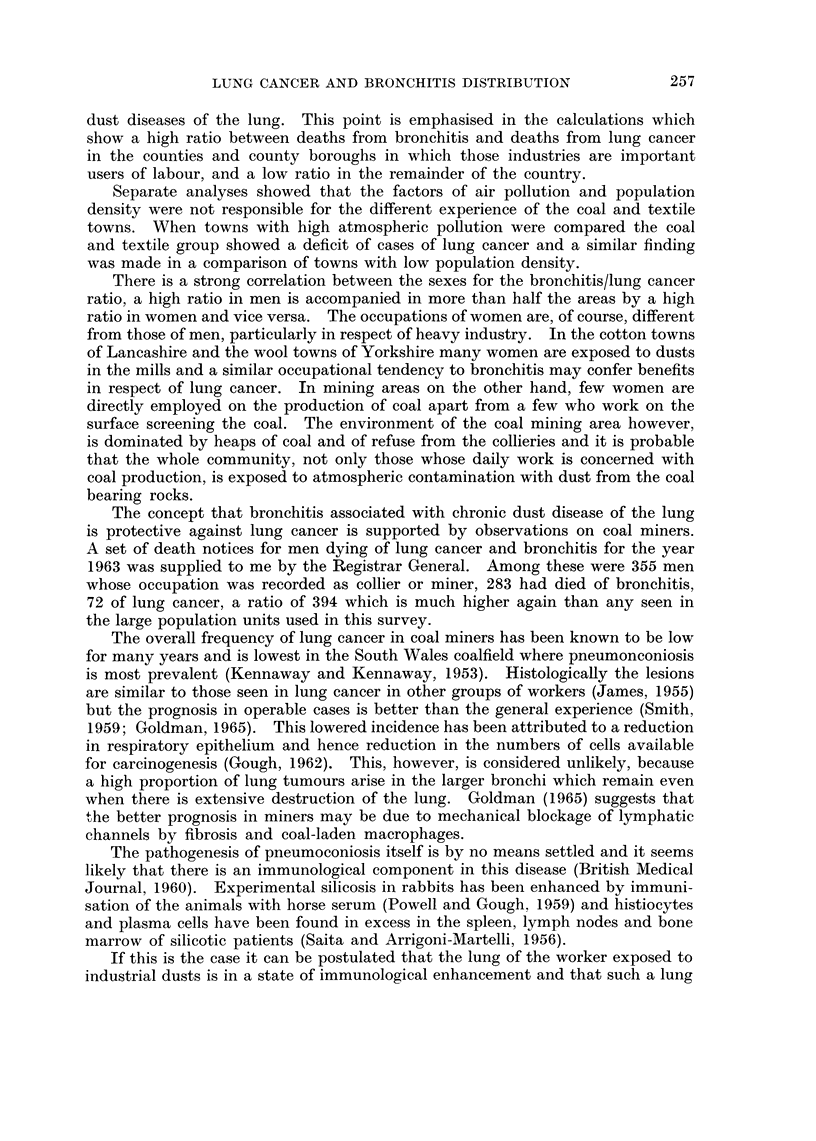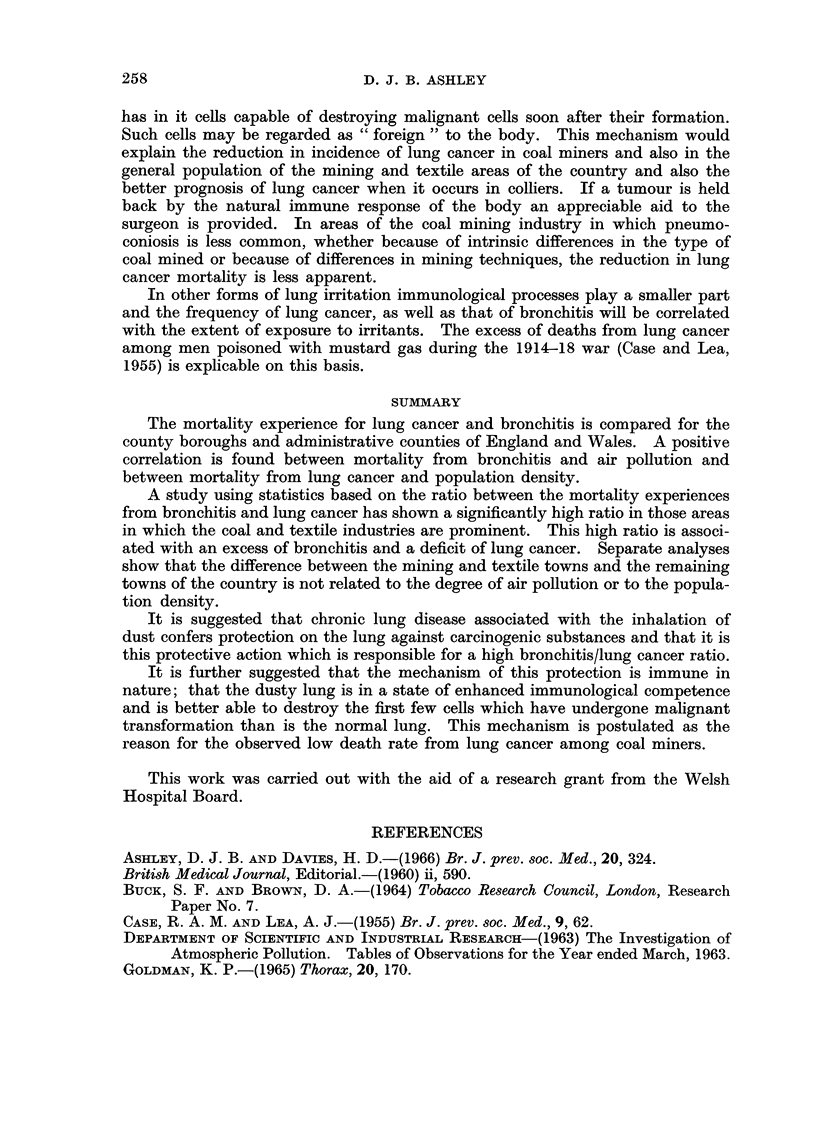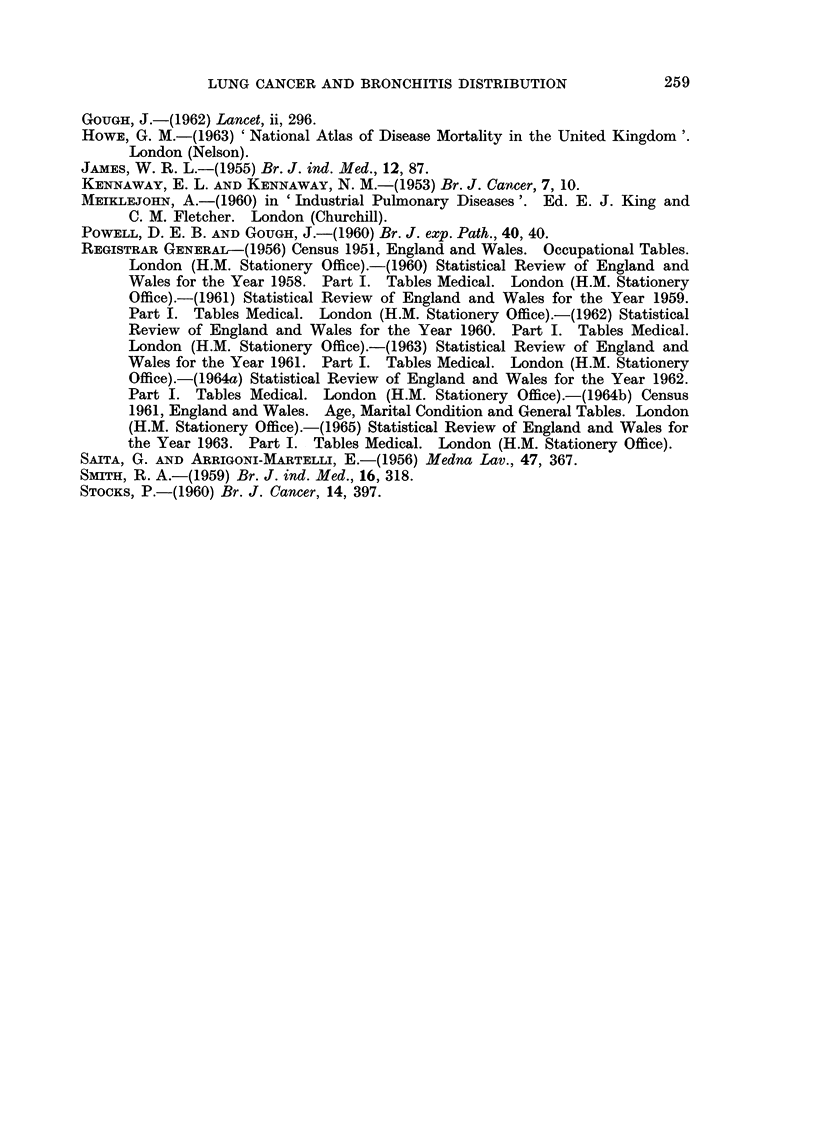# The distribution of lung cancer and bronchitis in England and Wales.

**DOI:** 10.1038/bjc.1967.27

**Published:** 1967-06

**Authors:** D. J. Ashley


### BRITISH JOURNAL OF CANCER

VOL. XXI JUNE, 1967 NO. 2

### THE DISTRIBUTION OF LUNG CANCER AND BRONCHITIS IN ENGLAND AND WALES

D. J. B. ASHLEY

*From the Morriston Hospital, Swansea*

Received for publication December 1, 1966

In the course of a recent study of the relationship between Welshness, indicated by the possession of a Welsh surname, and disease, it was noted that the death rate for lung cancer in men was low, S.M.R. 85, while that for bronchitis was high, S.M.R. 120, in the Registrar General's Standard Region Wales I which comprises the counties of Brecknock, Carmarthen, Glamorgan and Monmouth (Registrar General, 1965). The previous investigation (Ashley and Davies, 1966) was directed to testing and disproving the hypothesis that these differences were due to an inherently greater susceptibility to bronchitis and resistance to pulmonary neoplasm among the Welsh people. The investigation did, however, suggest that the form of bronchitis seen in coal miners might be to some extent protective against carcinogenic factors reaching the lung.

The present investigation extends to cover the whole of England and Wales and is based on statistics published in the Registrar General's Annual Reports for the years 1958 to 1963 (1960, 1961, 1962, 1963, 1964*a*, 1965). The mortality experience both for lung cancer and for chronic bronchitis is different in the urban and rural parts of the country and is directly correlated with the size of each town (Table I). For this reason a modified Standardised Mortality Ratio was used.

TABLE I.—*Standardised Mortality Ratios for Men in the Urban and Rural Parts of England and Wales.* 1963

| | Standardised mortality ratio | | |
|---|---|---|---|
| | Lung cancer | Bronchitis | Ratio of S.M.R.s |
| Conurbations | 122 | 121 | 99 |
| Urban areas of more than 100,000 | 112 | 117 | 104 |
| Urban areas of 50–100,000 | 96 | 92 | 96 |
| Urban areas of less than 50,000 | 86 | 90 | 104 |
| Rural areas | 72 | 69 | 97 |

The observed number of deaths from lung cancer and from bronchitis in each of the County Boroughs of England and Wales was extracted from the Annual Reports for the years 1958 to 1963. The expected number of deaths was calculated from the population distribution given in the Census Data of 1961 (Registrar General, 1964*b*) using the total death rates for the 6 year period for each age grouping in the three categories, conurbations, towns of over 100,000 population and towns of 50,000–100,000 population, calculated from the six Annual Reports (Table II) according to whether the county borough formed part of a conurbation

TABLE II.—*Death Rates per Thousand for Men for the Years 1958–63*

*Bronchitis*

| Age | 0– | 5– | 15– | 25– | 45– | 65– |
|---|---|---|---|---|---|---|
| Conurbations | 1·0 | 0·023 | 0·044 | 0·235 | 8·4 | 54·9 |
| Urban areas of over 100,000 | 0·865 | 0·057 | 0·040 | 0·220 | 6·45 | 39·5 |
| Urban areas of 50–100,000 | 0·85 | 0·037 | 0·027 | 0·160 | 5·78 | 36·0 |

*Lung Cancer*

| Age | 0– | 5– | 15– | 25– | 45– | 65– |
|---|---|---|---|---|---|---|
| Conurbations | — | — | — | 0·7 | 12·55 | 30·90 |
| Urban areas of over 100,000 | — | — | — | 0·499 | 9·65 | 23·18 |
| Urban areas of 50–100,000 | — | — | — | 0·573 | 9·15 | 21·02 |

TABLE III.—*Data from 84 Major Urban Areas in England and Wales*

| | Standardised mortality ratio males | | Ratio of S.M.R.s Male Bronchitis/Cancer | Ratio of deaths | | Population density | Air pollution | |
|---|---|---|---|---|---|---|---|---|
| | Lung cancer | Bronchitis | | Male | Female | | Smoke | $SO_2$ |
| London | 114* | 101* | 89* | 103 | 265 | — | — | — |
| Barnsley | 71* | 114 | 161* | 203 | 990 | 9·5 | 191 | 118 |
| Barrow | 128 | 80* | 62·5* | 71 | 196 | 5·9 | — | — |
| Bath | 98 | 83* | 85 | 98 | 244 | 12·9 | 42 | 44 |
| Birkenhead | 106 | 102 | 96 | 112 | 217 | 16·4 | 73 | 86 |
| Birmingham | 102* | 102* | 100 | 113 | 367 | 21·5 | — | — |
| Blackburn | 94* | 148* | 157* | 182 | 555 | 13·0 | 214 | 143 |
| Blackpool | 100* | 95* | 95 | 114 | 242 | 17·4 | 170 | 174 |
| Bolton | 84* | 99* | 118* | 139 | 467 | 10·5 | 158 | 196 |
| Bootle | 116 | 139* | 120 | 133 | 369 | 26·8 | 172 | 233 |
| Bournemouth | 101 | 67* | 66* | 85 | 171 | 13·1 | 61 | 102 |
| Bradford | 90* | 95* | 106 | 121 | 310 | 11·6 | 191 | 203 |
| Brighton | 99* | 95* | 96 | 97 | 171 | 11·3 | — | — |
| Bristol | 110 | 102* | 93 | 104 | 308 | 16·5 | 55 | 78 |
| Burnley | 104 | 125 | 120* | 147 | 440 | 17·1 | 141 | 181 |
| Burton | 92* | 137* | 149* | 166 | 379 | 12·0 | — | — |
| Bury | 70* | 114 | 163* | 188 | 551 | 8·0 | 232 | 213 |
| Canterbury | 99 | 88* | 89 | 103 | 254 | 6·5 | — | — |
| Carlisle | 118 | 91* | 77* | 83 | 277 | 11·6 | — | — |
| Chester | 124 | 120 | 97 | 107 | 256 | 12·7 | 103 | 100 |
| Coventry | 100* | 95* | 95 | 102 | 247 | 15·9 | 104 | 116 |
| Croydon | 102* | 79* | 77·5* | 92 | 273 | 19·8 | 55 | 124 |
| Darlington | 119 | 119 | 100 | 107 | 175 | 13·0 | — | — |
| Derby | 114 | 129* | 113 | 132 | 266 | 16·2 | 131 | 151 |
| Dewsbury | 82* | 119 | 145* | 184 | 675 | 7·8 | — | — |
| Doncaster | 88* | 119* | 135* | 150 | 516 | 10·2 | — | — |
| Dudley | 81* | 106 | 131* | 150 | 620 | 14·5 | — | — |
| Eastbourne | 112 | 68* | 61* | 78 | 120 | 5·5 | 56 | 44 |
| East Ham | 116 | 84* | 72·5* | 83 | 241 | 31·7 | — | — |
| Exeter | 100 | 91* | 91 | 104 | 309 | 8·8 | 50 | 54 |
| Gateshead | 119 | 110 | 92·5 | 107 | 308 | 22·6 | 79 | 51 |
| Gloucester | 112 | 127 | 113 | 125 | 317 | 13·2 | — | — |

| | Standardised mortality ratio males | | Ratio of of S.M.R.s male Bronchitis/ Cancer | Ratio of deaths | | Population density | Air pollution | |
|---|---|---|---|---|---|---|---|---|
| | Lung cancer | Bronchitis | | Male | Female | | Smoke | $SO_2$ |
| Great Yarmouth | 132 | 84* | 64* | 74 | 200 | 14·2 | — | — |
| Grimsby | 133* | 123 | 92·5 | 105 | 273 | 16·3 | — | — |
| Halifax | 90* | 91* | 101 | 121 | 367 | 6·8 | 110 | 94 |
| Hastings | 125 | 83* | 66·5* | 86 | 276 | 9·1 | — | — |
| Huddersfield | 75* | 91* | 121* | 141 | 293 | 9·2 | 166 | 191 |
| Ipswich | 99 | 64* | 65* | 77 | 155 | 11·7 | 98 | 95 |
| Kingston on Hull | 145* | 142* | 98 | 111 | 315 | 20·9 | 144 | 123 |
| Leeds | 104* | 118* | 113·5* | 130 | 315 | 12·5 | 153 | 188 |
| Leicester | 107 | 108 | 101 | 115 | 343 | 16·0 | 81 | 90 |
| Lincoln | 125 | 108 | 86 | 99 | 255 | 10·2 | 85 | 124 |
| Liverpool | 122* | 129* | 106* | 121 | 326 | 26·6 | 113 | 235 |
| Manchester | 124* | 154* | 124* | 141 | 452 | 24·1 | 140 | 177 |
| Middlesbrough | 146* | 118 | 81* | 90 | 314 | 21·9 | 131 | 97 |
| Newcastle | 119* | 100* | 84* | 98 | 267 | 24·3 | 172 | 119 |
| Northampton | 109 | 100 | 92 | 108 | 237 | 16·9 | — | — |
| Norwich | 118 | 86* | 73* | 87 | 205 | 14·7 | 80 | 80 |
| Nottingham | 133* | 170* | 128* | 143 | 425 | 16·9 | 94 | 50 |
| Oldham | 78* | 141* | 181* | 216 | 858 | 17·9 | 261 | 209 |
| Oxford | 108 | 86* | 80* | 90 | 177 | 12·2 | 57 | 148 |
| Plymouth | 104 | 77* | 74* | 87 | 286 | 16·2 | 30 | 33 |
| Portsmouth | 120 | 113 | 94 | 113 | 270 | 24·2 | 49 | 50 |
| Preston | 126* | 136* | 108 | 121 | 443 | 17·7 | — | — |
| Reading | 100 | 95* | 95 | 110 | 284 | 13·3 | 36 | 42 |
| Rochdale | 72* | 108 | 150* | 178 | 467 | 9·0 | — | — |
| Rotherham | 135* | 164* | 121·5* | 134 | 388 | 9·2 | — | — |
| St. Helens | 83* | 114 | 137* | 160 | 415 | 12·2 | 55 | 161 |
| Salford | 128* | 186* | 145* | 166 | 573 | 29·6 | 231 | 277 |
| Sheffield | 135* | 147* | 109* | 123 | 358 | 12·4 | 157 | 146 |
| Smethwick | 107 | 113 | 105 | 123 | 496 | 27·4 | 121 | 174 |
| Southampton | 126* | 105 | 83·5* | 97 | 235 | 17·7 | 66 | 73 |
| Southend | 120 | 81* | 67·5* | 84 | 190 | 16·0 | — | — |
| Southport | 118 | 121 | 103 | 125 | 250 | 8·4 | — | — |
| South Shields | 95* | 87* | 91·5 | 110 | 297 | 22·5 | 126 | 120 |
| Stockport | 92* | 111 | 121* | 140 | 367 | 16·8 | 184 | 176 |
| Stoke | 130* | 136* | 105 | 115 | 401 | 12·4 | — | — |
| Sunderland | 130* | 146* | 112 | 129 | 344 | 22·0 | 186 | 126 |
| Tynemouth | 113 | 76* | 67* | 80 | 206 | 14·9 | — | — |
| Wakefield | 77* | 119 | 155* | 182 | 430 | 10·4 | 202 | 181 |
| Wallasey | 103 | 76* | 74* | 88 | 191 | 17·4 | — | — |
| Walsall | 98* | 111 | 113* | 129 | 334 | 13·4 | 153 | 198 |
| Warrington | 131* | 177* | 135* | 130 | 360 | 16·7 | 202 | 226 |
| West Bromwich | 72* | 124 | 172* | 139 | 540 | 13·3 | 128 | 172 |
| West Ham | 162* | 145* | 90 | 99 | 256 | 33·4 | — | — |
| West Hartlepool | 138* | 153* | 111 | 123 | 487 | 16·4 | — | — |
| Wigan | 93* | 150* | 161* | 180 | 542 | 15·4 | — | — |
| Wolverhampton | 85* | 100* | 118* | 136 | 357 | 16·4 | — | — |
| Worcester | 131 | 131 | 100 | 111 | 731 | 10·8 | — | — |
| York | 116 | 111 | 96 | 109 | 185 | 15·2 | 132 | 121 |
| Cardiff | 117 | 123* | 105 | 121 | 403 | 17·0 | 34 | 61 |
| Merthyr Tydfil | 87* | 149* | 171* | 193 | 325 | 3·3 | — | — |
| Newport | 121 | 110 | 91 | 102 | 251 | 13·9 | 33 | 65 |
| Swansea | 94* | 120 | 128* | 146 | 442 | 7·7 | 23 | 42 |

* Indicates a significant difference from 110 in the case of S.M.R., 100 in the case of the Ratio of S.M.Rs.

or not and if not to its size. The modified Standardised Mortality Ratios and the ratio between them are set out in Table III for the 84 county boroughs. The combined totals for the county boroughs are; for carcinoma of lung 49,325 observed and 44,979 expected and for bronchitis 57,465 observed and 51,816 expected; giving S.M.R.s of 110 both for lung cancer and bronchitis. This finding is not unexpected as the urban areas used by the Registrar General in his calculations include many smaller municipalities which have not attained county borough status in which the incidence of these two conditions might be expected to be low. The standard errors of the calculated Standardised Mortality Ratio were determined and these which differed significantly from 110 are marked in the table with an asterisk.

The standardised mortality ratios for lung cancer and for chronic bronchitis were compared in the 84 boroughs by Spearman's Rank Correlation method. There was a significant positive correlation between the two ratios ($2\propto < 0{\cdot}01$) the value of the correlation coefficient was $+ 0{\cdot}3$.

An additional statistic which was calculated was the ratio between the Standardised Mortality Ratio for bronchitis and that for carcinoma. For the whole group of county boroughs this ratio was 100. As the two S.M.R.s were expected to be equal the significance of a difference from 100 in the ratio was determined indirectly by determining whether the two S.M.R.s for each county borough were significantly different. The difference between the two was regarded as significant if it exceeded twice the standard error of the difference between the two S.M.R.s *i.e.*

$$\mathrm{SMR_B} \sim \mathrm{SMR_C} > 2\sqrt{\frac{\mathrm{SMR_B}}{\mathrm{Exp_B}} + \frac{\mathrm{SMR_C}}{\mathrm{Exp_C}}}$$

The ratios which differed significantly from 100 are marked in the table with an asterisk. This ratio was selected for study because both lung cancer and bronchitis are associated with similar aetiological factors, among which smoking and air pollution are prominent and there is little urban/rural difference in the ratio. Limited factors, environmental or genetic, which might predispose selectively to one or the other of these diseases might escape detection if the mortality experience for each disease was considered separately. The ratio between the two provides an index of some sensitivity for such factors.

The next two columns in Table III show the ratios between the actual numbers of deaths from lung cancer and from bronchitis in males and females respectively. The correlation between the ratio of the S.M.R.s and the ratio of the actual number of deaths for males for each town was calculated by the rank correlation method. A highly significant positive correlation was found between these two estimates of the relationship between deaths from these two causes ($r = 0{\cdot}98$ $2\propto < 0{\cdot}001$).

The sixth column of Table III shows the population density of each town as calculated by the Registrar General from the Census data of 1961 (Registrar General, 1964*b*) and the final two columns show the concentration of smoke and of sulphur dioxide in the atmosphere at testing sites in residential areas of 53 county boroughs in respect of which this information was available (Department of Scientific and Industrial Research, 1963).

The ratio between the S.M.R.s for bronchitis and lung cancer may be influenced either by a change in the frequency of bronchitis or in the frequency of lung cancer or by both. Rank correlation calculations were carried out to compare the ratio of the S.M.R.s for bronchitis and lung cancer with the S.M.R. for bronchitis and with the S.M.R. for lung cancer separately. There was a significant negative correlation between this ratio and the S.M.R. for lung cancer ($r = -0{\cdot}31$ : $2\alpha < 0{\cdot}01$) and a highly significant positive correlation between the ratio and the S.M.R. for bronchitis ($r = 0{\cdot}733$ : $2\alpha < 0{\cdot}001$).

The ratio was significantly high in 29 County Boroughs and was significantly low in 19. The boroughs showing a low ratio, a relative deficit of bronchitis or an excess of carcinoma lay predominantly in the southern and south eastern part of England while those with a high ratio, a relative deficit of carcinoma or an excess of bronchitis lay predominantly in Lancashire and Yorkshire and in the Midlands. The reasons for the deviation from the expected ratio of 100 differ in the two groups of county boroughs. In the majority of those with a low ratio it could be related to a low mortality from bronchitis, in 17 of these towns the S.M.R. for bronchitis was significantly low while in only 4 was the S.M.R. for lung cancer raised, in 1 it was low and in the remaining 14 did not differ significantly from the overall mean (Table IV). The inference to be drawn is that in

TABLE IV.—*Standardised Mortality Rates for Lung Cancer and Bronchitis in the County Boroughs with a High and a Low Ratio between the S.M.R.s*

(a) Towns with a low ratio between the S.M.R.s

| | | S.M.R. Bronchitis | | | |
|---|---|---|---|---|---|
| | | High | Not significant | Low | |
| S.M.R. Lung cancer | High | — | 2 | 2 | 4 |
| | Not significant | — | — | 14 | 14 |
| | Low | — | — | 1 | 1 |
| | | — | 2 | 17 | 19 |

(b) Towns with a high ratio between the S.M.R.s

| | | S.M.R. Bronchitis | | | |
|---|---|---|---|---|---|
| | | High | Not significant | Low | |
| S.M.R. Lung cancer | High | 7 | — | — | 7 |
| | Not significant | — | 1 | — | 1 |
| | Low | 6 | 12 | 3 | 21 |
| | | 13 | 13 | 3 | 29 |

these areas the environmental conditions are not conducive to the development of bronchitis while carcinogenic factors, largely cigarette smoking, are fully operative. No less than 12 of these 19 boroughs are coastal towns while only 3 of the 29 towns with a high ratio are situated on the coast.

In the 29 towns with a high ratio the explanation is more complex. The S.M.R. for bronchitis was high in 13 instances and significantly low in 3 while the S.M.R. for lung cancer was high in seven boroughs and low in 21. Both ratios were high in the towns of Liverpool, Manchester, Nottingham, Rotherham, Salford, Sheffield, and Warrington. Four of these, Liverpool, Manchester, Nottingham and Sheffield are large cities which are noted for the presence in the recent past of a high degree of atmospheric pollution; Rotherham is almost contiguous with Sheffield and Salford with Manchester. In these cases it is reasonable to suggest that the high mortality from both lung cancer and bronchitis is related to environmental factors of a general nature which are directly related to the state of the atmosphere and which may be controlled by the current clean air programme.

In the other, larger, group in which the mortality from lung cancer was below expectation, some 14 of the 21 towns concerned were areas in which the coal mining and the cotton textile industries are of major importance. In 4 of these 14 towns there was a high mortality from bronchitis, in 2 there was a low mortality from bronchitis and in the remaining 8 the mortality experience from this cause did not differ significantly from that of the country as a whole.

These observations suggest that, in some areas of the country, there are environmental factors which are associated concomitantly with a decrease in the incidence of lung cancer and an excessive incidence of bronchitis. The previous observation (Ashley and Davies, 1966) of an excess of deaths from bronchitis and a deficiency of deaths from lung cancer in the South Wales coal mining area together with the finding that 14 of the 21 towns in which the ratio bronchitis S.M.R./lung cancer S.M.R. was high and the lung cancer S.M.R. was low were towns in which the coal industry and the textile industries were important employers of labour led to a specific analysis of the data from the 24 county boroughs in which these industries are prominent.

The towns selected for analysis were chosen on the basis of data given in the Occupation Tables for the Census of 1951 (Registrar General, 1956) as the occupation tables for the 1961 Census were not available. In 24 towns more than 4 per cent of the male population over the age of 15 years were employed in coal mining or in the textile industries (Table V).

TABLE V.—*Standardised Mortality Ratios for Lung Cancer and Bronchitis in 24 Coal Mining and Textile Towns*

| *Coal* | S.M.R. | | *Wool* | S.M.R. | | *Cotton* | S.M.R. | |
|---|---|---|---|---|---|---|---|---|
| | Lung cancer | Bronchitis | | Lung cancer | Bronchitis | | Lung cancer | Bronchitis |
| Barnsley | 71 | 114 | Bradford | 90 | 95 | Blackburn | 94 | 148 |
| Burnley | 94 | 148 | Dewsbury | 82 | 119 | Bolton | 84 | 99 |
| Dewsbury | 82 | 119 | Halifax | 90 | 91 | Burnley | 104 | 125 |
| Doncaster | 88 | 119 | Huddersfield | 75 | 91 | Bury | 70 | 114 |
| Gateshead | 119 | 110 | | | | Oldham | 78 | 141 |
| Nottingham | 133 | 170 | | | | Preston | 126 | 136 |
| Rotherham | 135 | 164 | | | | Rochdale | 72 | 108 |
| St. Helens | 83 | 114 | | | | Stockport | 92 | 111 |
| South Shields | 95 | 87 | | | | | | |
| Stoke | 130 | 136 | | | | | | |
| Sunderland | 130 | 146 | | | | | | |
| Wakefield | 77 | 119 | | | | | | |
| Wigan | 93 | 150 | | | | | | |
| Merthyr Tydfil | 87 | 149 | | | | | | |

In 14 of these towns coal mining was an important industry, in 8 the cotton textile industry and in 4 the wool textile industry. In some cases both mining and one of the textile industries coexisted.

The Standardised Mortality ratios for these 24 towns taken as a group are 98·7 for lung cancer and 121·2 for bronchitis. The ratio between the S.M.R.s is

123. There is, in the whole group a lower expectation of death from lung cancer and a higher expectation of death from chronic bronchitis.

Among the 14 mining towns the ratio between bronchitis and lung cancer was low in 2, Gateshead and South Shields, and was not significantly different from 100 in Stoke-on-Trent and Sunderland. In the remaining 10 towns the ratio was significantly raised. Nine of these towns had a significantly low S.M.R. for lung cancer, 4, Nottingham, Rotherham, Stoke and Sunderland had a significantly high S.M.R. and in 1, Gateshead, the S.M.R. did not differ significantly from that for the whole group of towns. The S.M.R. for bronchitis was high in 7 instances, low in 1 and did not differ significantly from the mean in the remaining 6. Taking the whole group together the S.M.R. for lung cancer was 109 which does not differ significantly from the general experience while that for bronchitis was 134 which is significantly raised. The ratio between the two S.M.R.s was 123.

Four of these five towns with a high S.M.R. for lung cancer however are in two divisions of the National Coal Board in which the frequency of pulmonary dust disease is low (Meiklejohn, 1960). Gateshead and Sunderland are in the North division and Nottingham and Rotherham in the East Midlands division. If these 4 are excluded the S.M.R. for lung cancer in the remaining 10 mining towns is 97·1 which is below the national average.

The pattern in the 8 cotton towns was more consistent. In 6, the S.M.R. for lung cancer was low, in 1, Preston, it was high and in 1 was not significantly lower than the average. In 3 towns the S.M.R. for bronchitis was high, in 1, Bolton, it was low and in the remaining 4 did not differ significantly from the average. The ratio between the S.M.R.s for bronchitis and for lung cancer was significantly raised in all but one town. The overall S.M.R.s were 89 for lung cancer and 121 for bronchitis, below and above the national average respectively.

The 4 county boroughs in which the wool industry assumed major importance all showed low S.M.R.s for lung cancer and all except Dewsbury, which is also a mining town, showed a low S.M.R. for bronchitis. The ratio was significantly raised in Dewsbury and in Huddersfield and did not differ significantly from the average in the cases of Bradford and Halifax. The overall S.M.R.s were 86·5 for lung cancer and 96·5 for bronchitis.

### *Air Pollution*

It has been suggested (Stocks, 1960) that the frequency of deaths from lung cancer and bronchitis may be related to the degree of atmospheric pollution in the homes and work places of different parts of the country. Buck and Brown (1964) however found a strong correlation between air pollution and bronchitis deaths but no such correlation with the frequency of deaths from lung cancer. The data from the County Boroughs were examined from this point of view with particular reference to the degree of air pollution in the coal and textile towns.

Data were available from testing stations in residential parts (Site Classification B) of 53 county boroughs in the National Survey of Air Pollution for March, 1963 (Department of Scientific and Industrial Research, 1963). Data from this single months were used as they were available for a large number of county boroughs and because Buck and Brown (1964) had shown an extremely strong correlation (+ 0·95) with the values for March, 1962 and the corresponding yearly averages where these were available. The concentrations of smoke and of sulphur dioxide in the air of these 53 towns were compared with the S.M.R.s for lung cancer and for bronchitis determined after allowance for the size of the towns had been made as above. The method of rank correlation was used throughout (Table III and VI). There was a very highly significant positive correlation

TABLE VI.—*Correlation Between Atmospheric Pollution, Lung Cancer and Bronchitis*

| | Coefficient | Significance |
|---|---|---|
| Smoke Concentration: $SO_2$ Concentration | +0·800 | Highly significant |
| Smoke Concentration: S.M.R. Lung cancer | −0·156 | Not significant |
| $SO_2$ Concentration: S.M.R. Lung cancer | −0·152 | Not significant |
| Smoke Concentration: S.M.R. Bronchitis | +0·494 | Highly significant |
| $SO_2$ Concentration: S.M.R. Bronchitis | +0·436 | Highly significant |

between the concentrations of smoke and sulphur dioxide and also between the S.M.R. for bronchitis and the concentrations both of smoke and of sulphur dioxide. There was a small, non-significant, negative correlation between the S.M.R. for lung cancer and the several concentrations of smoke and sulphur dioxide. These findings confirm those of Buck and Brown.

In the 53 towns the concentration of smoke varied from 23 to 261 $\mu g/m^3$ with a median of 124 and the concentrations of sulphur dioxide ranged from 33–277 again with a median value of 124. Sixteen towns in which coal mining and the textile industries were prominent, were included in this survey, 12 of these had atmospheric smoke concentrations above the median and 11 had sulphur dioxide concentrations above the median.

A separate analysis of the mortality experience in these towns which had, in March, 1963, a smoke concentration of more than 130 was made. Twenty four towns fell into this category, 11 were coal and textile towns, 13 were not: this is a significant excess of coal and textile towns. Nineteen towns had an S.M.R. for bronchitis of over 110, 8 of these were coal and textile, 11 were not: this difference is not significant. Eleven towns had an S.M.R. for lung cancer of over 110, only one of these was a coal or textile town, the remaining 10 were not: this difference is significant. Overall S.M.R.s were calculated for the 11 coal and textile towns and the 13 remaining towns in this group (Table VII).

TABLE VII.—*Standardised Mortality Ratios for Lung Cancer and Bronchitis in 24 Towns with High Atmospheric Pollution*

| | Lung cancer | Bronchitis |
|---|---|---|
| All 24 towns | 111·3 | 127·2 |
| 11 coal and textile towns | 89·7 | 114·2 |
| 13 remaining towns | 121·7 | 133·6 |

The S.M.R. for lung cancer for the whole group was almost the same as that for the whole group of 84 county boroughs; this finding accords with the hypothesis that air pollution is not correlated with lung cancer. The S.M.R. for bronchitis was higher than that for the whole group of 84 county boroughs; this again accords with the positive correlation between air pollution and bronchitis. In the coal and textile towns with high atmospheric pollution, however, the overall S.M.R. for lung cancer was only 89·7 which is much lower than the overall average and the overall S.M.R. for bronchitis, 114·2 was lower than that for the whole group of towns with dirty air. In the remaining 13 towns the S.M.R. for lung cancer was high and that for bronchitis very high. A less detailed analysis of the 23 towns with a high concentration of sulphur dioxide gave similar results.

These findings support the suggestion that there is a factor present in the coal and textile towns which is to some extent protective against lung cancer and also raise the hypothesis that a relationship between lung cancer and air pollution may be obscured by the selectively low S.M.R. for this disease in the coal and textile towns.

A separate calculation of correlation between the concentrations of smoke and sulphur dioxide and the S.M.R. for lung cancer was made for the 37 towns which were not coal and textile towns and for which air pollution data were available. There was a small positive correlation between the smoke concentration and the S.M.R. for lung cancer (+ 0·216) and also a small positive correlation between the sulphur dioxide concentration and S.M.R. for lung cancer. Neither of these correlations was significantly different from zero and it may be concluded that a relationship between the degree of air pollution and the S.M.R. for lung cancer, if one exists, is obscured by the other contributory factors.

*Population Density*

The density with which the population is packed together in the towns was found by Buck and Brown (1964) to be positively correlated with the frequency with which lung cancer was observed as a cause of death. In the present investigation the density of population, expressed as persons per acre (p.p.a.) was extracted from the Registrar General's General Tables for the Census of 1961 (Registrar General, 1964*b*) for the 83 county boroughs excluding Greater London (Table III), and was compared, by the rank correlation method, with the S.M.R.s calculated as above, for lung cancer and bronchitis. There was a positive correlation ($r = +\ 0{\cdot}359$) between the population density and the S.M.R. for lung cancer; this was significant at the 1 per cent level. There was also a positive correlation ($r = +\ 0{\cdot}184$) between the population density and the S.M.R. for bronchitis but this was not statistically significant.

These findings, and those in the preceding section on air pollution, support the contention that there are factors associated with air pollution which influence the frequency of bronchitis and other factors associated with crowding which influence pulmonary carcinogenesis (Buck and Brown, 1964). The lower death rate from lung cancer in the coal and textile towns could therefore be related to a difference in population density in these areas compared with the remainder of the country.

The population densities of the county boroughs varied from 3·3 to 33·4 with a median of 14·7 p.p.a. In the 24 coal and textile towns the population density varied from 3·3 to 22·6 p.p.a. with a median value of 12·2. This finding suggested that population density might be of importance in determining the low S.M.R. from lung cancer in these towns. A total of 24 towns, 12 coal and textile towns and 12 others, had a low population density of less than 12 persons per acre. Data from these two groups were extracted and combined S.M.R.s for lung cancer and for bronchitis were calculated for the whole group of towns of low population density, for the 12 coal and textile towns and for the 12 other towns (Table VIII). The S.M.R. for bronchitis for the whole group, 107·8 was only a

TABLE VIII.—*Standardised Mortality Ratios for Lung Cancer and Bronchitis in 24 Towns with Low Population Density*

| | Standardised mortality ratio | |
|---|---|---|
| | Lung cancer | Bronchitis |
| 12 coal and textile towns . | 85·7 | 116·6 |
| 12 other towns . . . . | 108·1 | 96·5 |
| 24 towns . . . . . | 95·2 | 107·8 |

little below that for the total of 84 county boroughs, a finding which supports the lack of correlation between the population density and the death rate from bronchitis. The S.M.R. for lung cancer was well below the overall average. When the two groups of towns are considered separately it is seen that the coal and textile towns have a favourable experience in respect of lung cancer (S.M.R. 85·7) and an unfavourable experience in respect of bronchitis (S.M.R. 116·6) when compared with the towns in which these industries are not prominent. These findings further support the contention that there is some specific factor in the environment of the coal and textile towns which is active against lung cancer development.

As there was such a close correlation between the ratio of the number of deaths from bronchitis and the number of deaths from lung cancer and the ratio of the S.M.R.s from these two causes, the former ratio was calculated for females (Table III), and for males and females in the administrative county areas outside the county boroughs (Table IX). Fig. 1 shows the distribution of significantly high and low ratios of observed deaths from these two conditions in males in the counties and county boroughs of England and Wales. The standard error of the ratio between the observed numbers of deaths was determined empirically by dividing the ratio by the square root of the smaller number of deaths whether bronchitis or carcinoma and the ratio was regarded as significantly different from the overall ratio for the counties, 116 in the case of men, 266 in the case of women, if the difference was greater than twice this standard error. The ratio for men was significantly raised in 14 counties, Derbyshire, Durham, Hereford, Lancashire, Nottinghamshire, Shropshire, Staffordshire, Worcestershire, the West Riding of Yorkshire, Brecknock, Carmarthen, Glamorgan, Monmouth and Pembroke. Significantly low ratios were seen in 28 counties which together occupied the Southern and South Eastern part of the country with the addition of Westmorland, the North Riding of Yorkshire and Radnorshire (Fig. 1).

The ratios for females followed those for males quite closely. Analysis of the counties and county boroughs separately by Spearman's rank correlation method showed in each case there was a significant correlation between the ratio for men and that for women. Thirteen counties showed a high ratio for women, ten of these also showed a high ratio for men; 19 counties showed a low ratio for women, 17 of these also showed a low ratio for men. Thirteen county boroughs showed a high ratio for women, in only one instance, Stoke-on-Trent, was the ratio for men normal. In 21 towns the ratio for women was low, 17 of these showed also a low ratio for men.

Inspection of the map shows that the areas in which the ratio of deaths from bronchitis to deaths from lung cancer is high correspond to the areas of the country in which the coal mining industry and the textile industries are concentrated.

TABLE IX.—*Deaths from Bronchitis and Lung Cancer. Counties England and Wales.* 1958–63

| | Males Bronchitis/ Carcinoma | Females Bronchitis/ Carcinoma |
|---|---|---|
| Bedfordshire | 98 | 247 |
| Berkshire | 89 | 213 |
| Buckingham | 94 | 242 |
| Cambridge | 98·5 | 239 |
| Cheshire | 116·5 | 303 |
| Cornwall | 88 | 175 |
| Cumberland | 108 | 214 |
| Derbyshire | 150 | 394 |
| Devon | 92 | 176 |
| Dorset | 89 | 195 |
| Durham | 131·5 | 296 |
| Ely | 128 | 400 |
| Essex | 95·5 | 221 |
| Gloucester | 105 | 201 |
| Hampshire | 88 | 200 |
| Hereford | 154 | 246 |
| Hertford | 88 | 223 |
| Huntingdon | 71 | 267 |
| Kent | 94 | 216 |
| Lancashire | 138 | 409 |
| Leicester | 107 | 260 |
| Lincoln | | |
| Holland | 107 | 288 |
| Kesteven | 89 | 226 |
| Lindsey | 106 | 311 |
| Middlesex | 88 | 203 |
| Norfolk | 90 | 252 |
| Northants | 112 | 405 |
| Northumberland | 111 | 270 |
| Nottingham | 144 | 380 |
| Oxford | 78 | 202 |
| Peterborough | 93 | 294 |
| Rutland | 100 | 83 |
| Shropshire | 128 | 383 |
| Somerset | 98·5 | 237 |
| Staffordshire | 144·5 | 455 |
| Suffolk East | 79 | 131 |
| Suffolk West | 95 | 242 |
| Surrey | 86 | 173 |
| Sussex East | 79 | 152 |
| Sussex West | 80 | 164 |
| Warwickshire | 105 | 278 |
| Westmorland | 92 | 147 |
| Isle of Wight | 84 | 194 |
| Wiltshire | 92·5 | 284 |
| Worcester | 151 | 415 |
| Yorkshire | | |
| East Riding | 107 | 263 |
| North Riding | 94·5 | 235 |
| West Riding | 156 | 414 |
| Anglesey | 103 | 555 |
| Brecon | 187 | 535 |
| Caernarvon | 105 | 265 |
| Cardigan | 119 | 485 |
| Carmarthen | 142 | 420 |
| Denbigh | 116 | 398 |
| Flint | 115 | 282 |
| Glamorgan | 204 | 487 |
| Merioneth | 114 | 246 |
| Monmouth | 198 | 533 |
| Montgomery | 79 | 312 |
| Pembroke | 144 | 216 |
| Radnor | 120 | 300 |

This confirms, in a general way the findings in the preceding sections that there is a differential incidence of bronchitis and of lung cancer in these areas.

More specific consideration of the counties shows that in 13 coal mining is an important industry and in 3 the textile industry is important. (Registrar General, 1956). Two of the three textile counties have high ratios between the numbers of deaths from bronchitis and lung cancer; these, Lancashire and the West Riding of Yorkshire are the seat of industries concerned with the processing of raw

FIG. 1.—Showing the distribution of high and low bronchitis/lung cancer ratios in the counties and county boroughs of England and Wales.

textile material. The third, Leicestershire is more concerned with the manufacture of textile articles from material which has already been processed elsewhere. The higher ratio in Lancashire and the West Riding may, on the present hypothesis, be related to the dust evolved in the primary processing operations which is not so apparent in later manufacturing processes.

Nine of the 13 mining counties showed significantly high ratios. The ratio in the remaining 4 counties were within the range which might be expected by chance. Two of these, Northumberland and Leicestershire were in Coal Board Divisions which have a low incidence of pneumoconiosis (Meiklejohn, 1960), and in the other two, Warwickshire and Denbighshire, Warwickshire has the lowest frequency of pneumoconiosis in its division and has a low proportion of the population working in the mines while in Denbighshire the frequency of pneumoconiosis is about the national average (Meiklejohn, 1960).

### DISCUSSION

Consideration of the statistics for deaths from lung cancer and bronchitis confirm the findings of Buck and Brown (1964) that bronchitis is correlated with the degree of atmospheric pollution while lung cancer shows no such correlation and, on the other hand lung cancer is correlated with population density while bronchitis is not.

The use of the two statistics, the ratio of the S.M.R.s for bronchitis and for lung cancer and the ratio between the gross numbers of deaths from these causes in different parts of the country, has pointed out differences in the mortality of different parts of the country which were unexpected (Fig. 1). The mortality experience of different sizes of towns differs in these two diseases (Table I) and the calculated S.M.R.s make allowance for these differences but the ratio between the S.M.R.s (Table I) and between the gross numbers of deaths (Table X) do not show any significant differences in towns of different population.

TABLE X.—*Mortality Experience for Lung Cancer and Bronchitis Among Men in the Urban and Rural Parts of England and Wales.* 1963

| | Deaths | | |
|---|---|---|---|
| | Lung cancer | Bronchitis | Bronchitis/ Lung cancer |
| Conurbations | 8809 | 10,176 | 115 |
| Urban areas of more than 100,000 | 3040 | 3757 | 123 |
| Urban areas of 50–100,000 | 1858 | 2157 | 116 |
| Urban areas of less than 50,000 | 3820 | 4927 | 129 |
| Rural areas | 3230 | 3815 | 119 |
| | 20,757 | 24,832 | 120 |

A low ratio between the S.M.R.s was generally correlated with a low death rate from bronchitis and with a low degree of atmospheric pollution. This would be expected if the urban factors responsible for the higher incidence both of bronchitis and of lung cancer in the larger towns were independent and the correlation between the two coincidental. Areas with a high ratio, however, showed a more complex picture, in some instances there was an excessively high mortality experience from bronchitis while in others the mortality from lung cancer was unusually low.

A first simple hypothesis is that in areas of a high prevalence of bronchitis patients die of this condition before they have time to develop lung cancer. The age distribution of deaths from these two causes however (Tables II and XI) show that death from bronchitis, when it occurs, tends to involve men at a greater age than is the case for lung cancer; the relative numbers of deaths from the two causes shows a steady increase with increasing age.

A second hypothesis is that the differences in total deaths from bronchitis and lung cancer may be related to differing age structures in the population. This would involve an opposite age effect to that put forward in the preceding

TABLE XI.—*Bronchitis/Lung Cancer Ratio by Age. England and Wales* 1963

| | Males | | | | Females | | |
|---|---|---|---|---|---|---|---|
| | Deaths | | | | Deaths | | |
| | Carcinoma | Bronchitis | Ratio | | Carcinoma | Bronchitis | Ratio |
| 40– | 1182 | 501 | 42 | . | 331 | 221 | 67 |
| 50– | 4180 | 2958 | 71 | . | 823 | 671 | 82 |
| 60– | 8484 | 7825 | 92 | . | 1184 | 1857 | 173 |
| 70– | 4789 | 8657 | 180 | . | 942 | 3652 | 389 |
| 80– | 880 | 4444 | 505 | . | 321 | 3754 | 1180 |

paragraph. Bronchitis is responsible for twice as many deaths in men over 70 than is lung cancer, while in men between 40 and 50 only half as many deaths from bronchitis occur as deaths from lung cancer. A population with a high proportion of older people would, therefore, be expected to have a relatively unfavourable experience in respect of bronchitis and consequently a high bronchitis/lung cancer ratio. The Standardised Mortality Ratios do not bear this out. When allowance is made for the age structure of the population there is still a difference between areas with a high ratio and those with a low ratio.

The point was further tested by comparing the ratio between the S.M.R.s with the proportion of the male population over the age of 65 years (Registrar General, 1964*b*) (Table XII). This table shows a tendency for the ratio of the

TABLE XII.—*Comparison of the Ratio Between the S.M.R.s for Lung Cancer and Bronchitis With the Proportion of Elderly Men in the Population*

| Ratio of S.M.R.s | | Proportion of men over 65 years | | |
|---|---|---|---|---|
| | | <4% | 4–5% | >5% |
| −80 | . | 0 | 8 | 6 |
| −110 | . | 8 | 23 | 6 |
| −140 | . | 8 | 13 | 0 |
| >140 | . | 2 | 9 | 0 |

S.M.R.s to be lower in the towns which have a high proportion of men over the age of 65. The towns concerned however, Bath, Blackpool, Bournemouth, Brighton, Eastbourne, Great Yarmouth, Hastings, Northampton, Norwich, Portsmouth, Southend and Southport are predominantly in coastal areas of the southern part of the country where there is generally little industry and the degree of air pollution is low.

The finding of an excess of deaths from bronchitis and a deficiency of deaths from lung cancer in the high ratio areas and the converse finding in the low ratio areas, suggests that bronchitis may have a protective effect on the lungs, and that the patient who has this chronic inflammatory and degenerative process at work may be at a relative advantage *vis-a-vis* his fellow with more normal lungs. The distribution of high and low bronchitis/lung cancer ratios (Fig. 1) does not correspond however to the distribution of deaths from bronchitis in the population (Howe, 1963). Areas such as Merseyside, London and Sheffield in which there is a high frequency of bronchitis do not appear to have a reduced frequency of lung cancer. The distribution of high ratio areas, those in which there is an excess of bronchitis and a deficiency of lung cancer, corresponds rather to the distribution of the coal and textile industries in which there are occupational dust diseases of the lung. This point is emphasised in the calculations which show a high ratio between deaths from bronchitis and deaths from lung cancer in the counties and county boroughs in which those industries are important users of labour, and a low ratio in the remainder of the country.

Separate analyses showed that the factors of air pollution and population density were not responsible for the different experience of the coal and textile towns. When towns with high atmospheric pollution were compared the coal and textile group showed a deficit of cases of lung cancer and a similar finding was made in a comparison of towns with low population density.

There is a strong correlation between the sexes for the bronchitis/lung cancer ratio, a high ratio in men is accompanied in more than half the areas by a high ratio in women and vice versa. The occupations of women are, of course, different from those of men, particularly in respect of heavy industry. In the cotton towns of Lancashire and the wool towns of Yorkshire many women are exposed to dusts in the mills and a similar occupational tendency to bronchitis may confer benefits in respect of lung cancer. In mining areas on the other hand, few women are directly employed on the production of coal apart from a few who work on the surface screening the coal. The environment of the coal mining area however, is dominated by heaps of coal and of refuse from the collieries and it is probable that the whole community, not only those whose daily work is concerned with coal production, is exposed to atmospheric contamination with dust from the coal bearing rocks.

The concept that bronchitis associated with chronic dust disease of the lung is protective against lung cancer is supported by observations on coal miners. A set of death notices for men dying of lung cancer and bronchitis for the year 1963 was supplied to me by the Registrar General. Among these were 355 men whose occupation was recorded as collier or miner, 283 had died of bronchitis, 72 of lung cancer, a ratio of 394 which is much higher again than any seen in the large population units used in this survey.

The overall frequency of lung cancer in coal miners has been known to be low for many years and is lowest in the South Wales coalfield where pneumonconiosis is most prevalent (Kennaway and Kennaway, 1953). Histologically the lesions are similar to those seen in lung cancer in other groups of workers (James, 1955) but the prognosis in operable cases is better than the general experience (Smith, 1959; Goldman, 1965). This lowered incidence has been attributed to a reduction in respiratory epithelium and hence reduction in the numbers of cells available for carcinogenesis (Gough, 1962). This, however, is considered unlikely, because a high proportion of lung tumours arise in the larger bronchi which remain even when there is extensive destruction of the lung. Goldman (1965) suggests that the better prognosis in miners may be due to mechanical blockage of lymphatic channels by fibrosis and coal-laden macrophages.

The pathogenesis of pneumoconiosis itself is by no means settled and it seems likely that there is an immunological component in this disease (British Medical Journal, 1960). Experimental silicosis in rabbits has been enhanced by immunisation of the animals with horse serum (Powell and Gough, 1959) and histiocytes and plasma cells have been found in excess in the spleen, lymph nodes and bone marrow of silicotic patients (Saita and Arrigoni-Martelli, 1956).

If this is the case it can be postulated that the lung of the worker exposed to industrial dusts is in a state of immunological enhancement and that such a lung has in it cells capable of destroying malignant cells soon after their formation. Such cells may be regarded as " foreign " to the body. This mechanism would explain the reduction in incidence of lung cancer in coal miners and also in the general population of the mining and textile areas of the country and also the better prognosis of lung cancer when it occurs in colliers. If a tumour is held back by the natural immune response of the body an appreciable aid to the surgeon is provided. In areas of the coal mining industry in which pneumoconiosis is less common, whether because of intrinsic differences in the type of coal mined or because of differences in mining techniques, the reduction in lung cancer mortality is less apparent.

In other forms of lung irritation immunological processes play a smaller part and the frequency of lung cancer, as well as that of bronchitis will be correlated with the extent of exposure to irritants. The excess of deaths from lung cancer among men poisoned with mustard gas during the 1914–18 war (Case and Lea, 1955) is explicable on this basis.

SUMMARY

The mortality experience for lung cancer and bronchitis is compared for the county boroughs and administrative counties of England and Wales. A positive correlation is found between mortality from bronchitis and air pollution and between mortality from lung cancer and population density.

A study using statistics based on the ratio between the mortality experiences from bronchitis and lung cancer has shown a significantly high ratio in those areas in which the coal and textile industries are prominent. This high ratio is associated with an excess of bronchitis and a deficit of lung cancer. Separate analyses show that the difference between the mining and textile towns and the remaining towns of the country is not related to the degree of air pollution or to the population density.

It is suggested that chronic lung disease associated with the inhalation of dust confers protection on the lung against carcinogenic substances and that it is this protective action which is responsible for a high bronchitis/lung cancer ratio.

It is further suggested that the mechanism of this protection is immune in nature; that the dusty lung is in a state of enhanced immunological competence and is better able to destroy the first few cells which have undergone malignant transformation than is the normal lung. This mechanism is postulated as the reason for the observed low death rate from lung cancer among coal miners.

This work was carried out with the aid of a research grant from the Welsh Hospital Board.

### REFERENCES

ASHLEY, D. J. B. AND DAVIES, H. D.—(1966) *Br. J. prev. soc. Med.*, **20**, 324.

*British Medical Journal*, Editorial.—(1960) ii, 590.

BUCK, S. F. AND BROWN, D. A.—(1964) *Tobacco Research Council, London*, Research Paper No. 7.

CASE, R. A. M. AND LEA, A. J.—(1955) *Br. J. prev. soc. Med.*, **9**, 62.

DEPARTMENT OF SCIENTIFIC AND INDUSTRIAL RESEARCH—(1963) The Investigation of Atmospheric Pollution. Tables of Observations for the Year ended March, 1963.

GOLDMAN, K. P.—(1965) *Thorax*, **20**, 170.

Gough, J.—(1962) *Lancet*, ii, 296.
Howe, G. M.—(1963) ' National Atlas of Disease Mortality in the United Kingdom '. London (Nelson).
James, W. R. L.—(1955) *Br. J. ind. Med.*, **12**, 87.
Kennaway, E. L. and Kennaway, N. M.—(1953) *Br. J. Cancer*, **7**, 10.
Meiklejohn, A.—(1960) in ' Industrial Pulmonary Diseases '. Ed. E. J. King and C. M. Fletcher. London (Churchill).
Powell, D. E. B. and Gough, J.—(1960) *Br. J. exp. Path.*, **40**, 40.
Registrar General—(1956) Census 1951, England and Wales. Occupational Tables. London (H.M. Stationery Office).—(1960) Statistical Review of England and Wales for the Year 1958. Part I. Tables Medical. London (H.M. Stationery Office).—(1961) Statistical Review of England and Wales for the Year 1959. Part I. Tables Medical. London (H.M. Stationery Office).—(1962) Statistical Review of England and Wales for the Year 1960. Part I. Tables Medical. London (H.M. Stationery Office).—(1963) Statistical Review of England and Wales for the Year 1961. Part I. Tables Medical. London (H.M. Stationery Office).—(1964*a*) Statistical Review of England and Wales for the Year 1962. Part I. Tables Medical. London (H.M. Stationery Office).—(1964b) Census 1961, England and Wales. Age, Marital Condition and General Tables. London (H.M. Stationery Office).—(1965) Statistical Review of England and Wales for the Year 1963. Part I. Tables Medical. London (H.M. Stationery Office).
Saita, G. and Arrigoni-Martelli, E.—(1956) *Medna Lav.*, **47**, 367.
Smith, R. A.—(1959) *Br. J. ind. Med.*, **16**, 318.
Stocks, P.—(1960) *Br. J. Cancer*, **14**, 397.